# Connaissances et attitudes des relais communautaires sur les fièvres hémorragiques à virus Lassa et Ebola dans le département de la Donga (Nord Bénin)

**DOI:** 10.11604/pamj.2017.26.229.12072

**Published:** 2017-04-25

**Authors:** Cossi Angelo Attinsounon, Cossi Athanase Hounnankan, Comlan Albert Dovonou, Cossi Adébayo Alassani, Sourakatou Salifou

**Affiliations:** 1 UER de Maladies Infectieuses et Tropicales, Faculté de Médecine, Université de Parakou, Bénin; 2 Projet d’Intensification de la Lutte contre le Paludisme, Ministère de la Santé, Bénin; 3 Service de Médecine Interne, CHUD Borgou-Alibori, Parakou, Bénin; 4 Zone Sanitaire Djougou Copargo Ouaké, Ministère de la santé, Bénin

**Keywords:** Attitude, connaissance, Ebola, Lassa, Bénin, relais communautaires, Afrique de l´Ouest, Attitude, knowledge, Ebola, Lassa, Benin, community volunteers, West Africa

## Abstract

**Introduction:**

L’objectif de cette étude était d’évaluer les connaissances et attitudes des relais communautaires vis-à-vis des fièvres hémorragiques à virus Ebola et Lassa et leur implication dans la mise œuvre des activités de prévention de ces maladies.

**Méthodes:**

Une enquête transversale descriptive a été menée auprès des relais communautaires recrutés par tirage au sort dans 40 villages du département de la Donga. Ces relais faisaient la prise en charge à domicile des maladies respiratoires, diarrhéiques et du paludisme chez les enfants de moins de cinq ans. Un questionnaire anonyme a été administré par interview directe. Les données ont été analysées à l’aide du logiciel Epi-info 3.5.1.

**Résultats:**

Au total 58 relais communautaires (RC) ont participé à cette enquête sur les 60 attendus. L’âge moyen était de 38,7±10,6 ans avec un sex-ratio de 3,5. Il y avait majoritairement trente cinq cultivateurs (60,3%) et treize revendeuses (22,4%). Quarante huit enquêtés (82,8%) reconnaissaient les deux maladies comme étant graves, mortelles et transmissibles. Les trois principales voies de transmission citées étaient le contact ou la consommation de gibiers (87,9%), le contact direct avec les personnes infectées (74,1%) ou leurs cadavres (46,6%). Les principaux moyens préventifs énumérés étaient en lien avec les voies de transmission. La fièvre (81,0%), les vomissements (81,0%) et la diarrhée (60,3%) venaient en tête des symptômes cités. Seulement vingt-deux RC (37,9%) disposaient de gants mais les utilisaient rarement pour examiner les enfants malades. Quant à la conduite à tenir devant un cas suspect de fièvre hémorragique virale Lassa ou Ebola, quarante-et-un relais communautaires (70,7%) feraient recours aux agents de santé sans toucher au malade, neuf (15,5%) feraient appel à l’ambulance et huit (13,8%) transporteraient le cas sur leur propre moto ou sur un taxi-moto vers le centre de santé le plus proche.

**Conclusion:**

Le renforcement des capacités des relais communautaires sur les fièvres hémorragiques virales contribuerait à l’amélioration de leurs connaissances sur ces épidémies mortelles et à la qualité de leurs interventions dans la population.

**Introduction:**

This study aimed to evaluate the knowledges and attitudes of community volunteers on Lassa and Ebola viral haemorrhagic fevers and their role in the implementation of activities for the prevention of these diseases.

**Methods:**

We conducted a cross-sectional descriptive survey among community volunteers chosen by lot in 40 villages in the Donga Department. Children under five years of age with respiratory and diarrheal diseases and malaria were treated by these community volunteers in their home. An anonymous questionnaire was administered by direct interview. Data were analyzed using Epi-Info 3.5.1.

**Results:**

Out of 60 community volunteers potentially participating in this survey a total of 58 effectively participated. The average age of community volunteers was 38.7 ± 10.6 years with a sex-ratio of 3.5. The majority of the community volunteers were farmers (thirty-five, 60.3%) and resellers (thirteen, 22.4%). Forty-eight respondents (82.8%) recognized the two diseases as being serious, life-threatening and transmissible. The three main routes of transmission cited were the contact with or the consumption of bushmeat (87.9%), the direct contact with infected people (74.1%) or with their corpses (46.6%). The main preventive measures listed were related to the routes of transmission. Fever (81.0%), vomiting (81.0%) and diarrhea (60.3%) were at the top of the cited symptoms. Only twenty-two community volunteers (37.9%) had gloves but they rarely used them to examine sick children. As regards the procedure to follow in case of suspected Lassa or Ebola viral haemorrhagic fever , forty-one community volunteers (70.7%) would make use of community-based health workers without touching the patient, nine (15.5%) would call for the ambulance and eight (13.8%) would take the patient to the nearest health center using their own motorcycle or a motorcycle-taxi.

**Conclusion:**

Strengthening community volunteers’ capacity to manage viral hemorrhagic fevers would contribute to the improvement of their knowledge of these life-threatening epidemics and to the quality of population health interventions.

## Introduction

A partir de mars 2014, l’Afrique de l’Ouest a connu la plus grande épidémie meurtrière de la maladie à virus Ebola [1]. Plusieurs dispositions à visée préventive et interventionniste ont été prises dans plusieurs pays [1, 2]. Comme pour tester le dispositif mis en place pour répondre efficacement à une éventuelle épidémie de maladie à virus Ebola, le Bénin a connu successivement deux épidémies de fièvre Lassa [3]. Le Bénin en effet fait partie des zones à risque de la fièvre hémorragique à virus Lassa [4-6]. Considérée comme endémique en milieu hospitalier où elle règne en tueuse des agents de santé dans les contextes de sécurité insuffisante aux soins, la fièvre Lassa peut aussi faire l’objet d’épidémie sporadique dans la population générale [3, 7, 8]. Le Bénin depuis 2009 a développé une stratégie de prise en charge intégrée des maladies de l’enfance en milieu communautaire (PCIME- Communautaire). Cette stratégie est mise en œuvre par les relais communautaires qui sont des personnes identifiées par leurs paires et formées à la promotion de la santé. A cet effet, ils mènent des activités de communication pour un changement de comportement d’une part et assure la prise en charge à domicile de la toux, de la diarrhée et du paludisme chez les enfants de moins de cinq ans d‘autre part. C’est dans ce cadre qu’ils ont bénéficié d’un briefing d’une demi-journée sur les fièvres hémorragiques virales (FVH) en vue de leur participation à la prévention de ces maladies dans leurs villages respectifs. Six mois après la fin de l’épidémie de fièvre Lassa au Bénin, nous avons mené cette étude en vue d’apprécier les changements comportementaux induits, les connaissances acquises et les performances de ces acteurs communautaires dans la lutte contre les fièvres hémorragiques virales.

## Méthodes

Il s’agissait d’une enquête transversale menée en juillet 2015 auprès des relais communautaires recrutés de façon systématique dans quarante (40) villages tirés au sort dans les trois communes (Djougou, Ouaké et Copargo) du département de la Donga au Nord-Bénin. Les relais communautaires sont des volontaires villageois identifiés par leurs pairs et formés sur la prise en charge des maladies des enfants de moins de 5 ans et sur la promotion de la santé dans leurs communautés. Dans le cadre de la prise en charge du paludisme à domicile, ces relais réalisent des tests de diagnostic rapide (TDR) du paludisme avant l’administration d’un antipaludique aux enfants ayant la fièvre. Ils sont par conséquent régulièrement victimes d’accidents d’exposition au sang sans être conscients de la gravité et du risque infectieux qu’ils encourent. Un questionnaire anonyme a été administré à travers une interview directe après le consentement de chaque enquêté. Le questionnaire prenait en compte trois catégories de données que sont les données sociodémographiques, celles relatives aux connaissances et aux attitudes des enquêtés. Les données ont été saisies et analysées à l’aide du logiciel Epi-info 3.5.1.

## Résultats

### Caractéristiques sociodémographiques des enquêtés

Au total cinquante huit (58) relais communautaires (RC) ont participé à cette enquête sur les 60 attendus soit un taux de participation de 96,7%. L’âge moyen était de 38,7±10,6 ans avec des extrêmes de 20 et 64 ans et le sex-ratio était de 3,5. Il s’agissait majoritairement de cultivateurs (60,3%) suivis de revendeuses (22,4%). La moitié avait un niveau primaire (**Tableau 1**).

**Tableau 1:** caractéristiques sociales et démographiques des cinquante-huit relais communautaires interviewés dans le département de la Donga (Nord – Bénin)

Paramètres		Effectifs	%
**Age (ans)**	20 - 40	41	70,7
	40 - 60	14	24,1
	> 60	03	05,2
	**Total**	**58**	**100,0**
**Genre**	Masculin	45	77,6
	Féminin	13	22,4
	**Total**	**58**	**100,0**
**Profession**	Cultivateurs	35	60,3
	Revendeuses	13	22,4
	Artisans	10	17,3
	**Total**	**58**	**100,0**
**Niveau d’instruction**	Primaire	29	50,0
	Secondaire	29	50,0
	**Total**	**58**	**100,0**

### Connaissances sur les maladies à virus Lassa et Ebola

**Figure 1:**
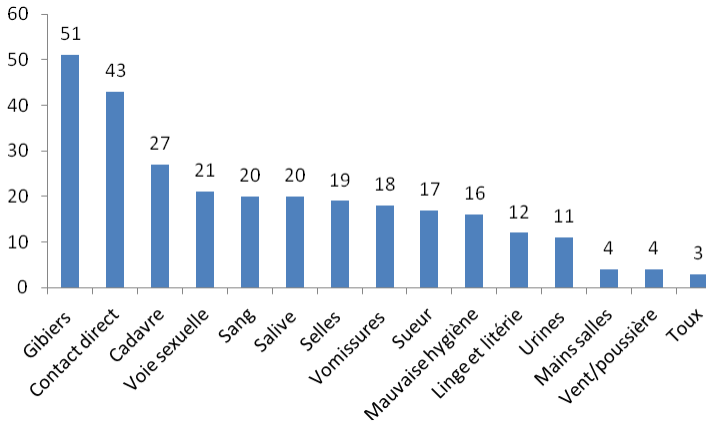
Modes de transmission des maladies à virus Lassa et Ebola cités par les cinquante-huit relais communautaires interviewés dans la Donga (Nord-Bénin)

**Figure 2:**
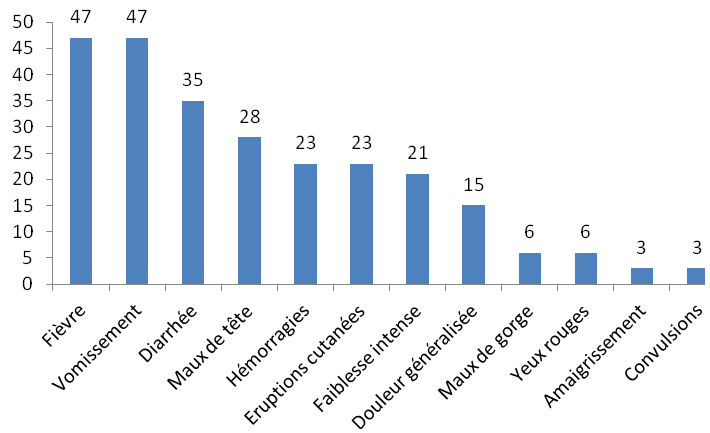
Manifestations cliniques des maladies à virus Lassa et Ebola citées par les cinquante-huit relais communautaires interviewés dans la Donga (Nord-Bénin)

**Figure 3:**
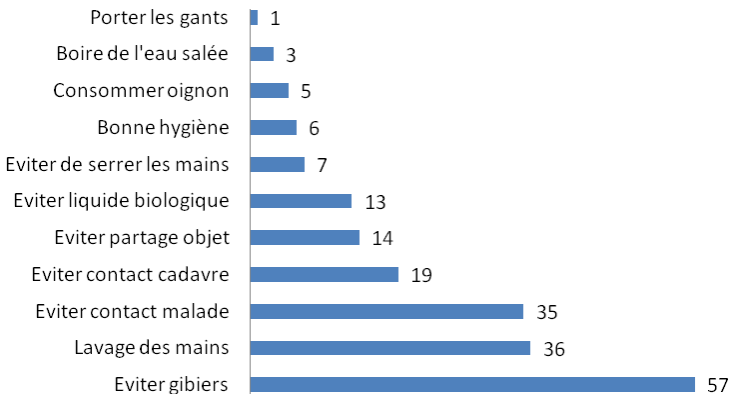
Moyens de prévention des maladies à virus Lassa et Ebola cités par les cinquante-huit relais communautaires interviewés dans la Donga (Nord-Bénin)

Quarante neuf relais (84,5%) avaient reçu une formation d’une demi-journée sur les FHV six mois avant l’enquête. Ces formations étaient conduites par les agents de santé de leurs aires sanitaires respectives. Tous les cinquante huit (100%) relais communautaires déclaraient avoir eu des informations sur les FHV Lassa et Ebola à travers les médias (radios et télévisions). Trente quatre relais (58,6%) ne savaient pas qu’il y avait eu une épidémie de fièvre Lassa au Bénin. Par contre, trois enquêtés (5,2%) estimaient que la maladie à virus Ebola était diagnostiquée au Bénin. Quarante huit relais (82,8%) reconnaissaient les deux maladies comme étant graves, mortelles et transmissibles. Les trois principales voies de transmission citées étaient le contact avec les animaux de brousse ou la consommation des gibiers (87,9%), le contact direct avec les sujets malades (74,1%) ou avec leurs cadavres (46,6%) (Figure 1). Aucun enquêté ne faisait le lien entre les rongeurs et la fièvre Lassa d’une part et entre les chauves-souris et la maladie à virus Ebola d’autre part. En ce qui concerne les manifestations cliniques des deux maladies, la fièvre (81,0%), les vomissements (81,0%), la diarrhée (60,3%) venaient en tête des symptômes cités par les RC (Figure 2). Les trois principaux moyens préventifs cités par les enquêtés étaient proportionnellement en lien avec les voies de transmission (Figure 3). Dix huit enquêtés (31,0%) déclaraient qu’il y avait un traitement curatif et un vaccin efficaces contre les virus Ebola et Lassa.

### Attitudes et rôles joués dans la prévention des maladies à virus Lassa et Ebola

Cinquante trois RC (91,4%) menaient des activités de sensibilisation sous forme de séance de causeries éducatives sur les FHV dans leur village. Les dernières séances remontaient à en moyenne 3,3 ± 2,0 mois avant le jour de l’enquête. Seulement vingt-deux RC (37,9%) disposaient de gants mais les utilisaient rarement pour examiner les enfants malades. Cinq RC (8,6%) utilisaient les gants seulement au moment de la réalisation des tests de diagnostic rapide du paludisme (TDR). Aucun d’eux ne disposait de blouse, tablier, solutions hydro-alcooliques, lunettes et masque de protection. Quant à la conduite à tenir devant un cas suspect de maladie à virus Ebola ou de fièvre Lassa, quarante-et-un RC (70,7%) feraient recours aux agents de santé sans toucher au malade, neuf (15,5%) feraient appel à l’ambulance de la zone sanitaire et huit (13,8%) transporteraient le cas sur leur propre moto (8,6%) ou sur un taxi-moto (5,2%) communément appelé « zémidjan » en langue nationale béninoise vers le centre de santé le plus proche.

## Discussion

Cette enquête était menée dans un contexte d’épidémie nationale de la maladie à virus Lassa et dans un contexte régional voire international de maladie à virus Ebola. La cible se justifiait par le fait qu’elle courait des risques infectieux liés aux soins car ils jouaient dans leurs communautés le rôle d’agent de santé. Cette étude nous a permis d’apprécier non seulement leurs connaissances sur les deux maladies, mais aussi, le rôle qu’ils jouaient dans la prévention des deux FHV et l’attitude qu’ils adopteraient en cas d’épidémie de maladie à virus Ebola au Bénin. Les relais connaissaient ou ont plus entendu parler de la maladie à virus Ebola que de la fièvre Lassa. Ils ignoraient dans 41% des cas qu’il y avait eu une épidémie de fièvre Lassa au Bénin mais par contre trois d’entre eux affirmaient qu’il y a eu plutôt des cas confirmés de maladie à virus Ebola au Bénin. C’est la preuve que l’épidémie de fièvre Lassa survenue au Bénin retenait moins d’attention dans la population béninoise et se serait plutôt noyée dans la phobie régionale provoquée par la maladie à virus Ebola. Les enquêtes menées auprès des agents de santé qualifiés par certains auteurs montraient une meilleure connaissance de la maladie à virus Ebola par rapport à la fièvre Lassa [9-12].

Du point de vue de leur ressemblance, environ 28% des relais savaient que les deux maladies avaient les mêmes modes de transmission interhumaine et les mêmes symptômes. Les relais avaient globalement une connaissance moyenne des modes de transmission des deux maladies. Ils ignoraient très souvent la contamination par les liquides biologiques alors qu’ils réalisaient tous à domicile des TDR pour le dépistage du paludisme chez les enfants. Ils ne maitrisaient pas les risques infectieux surtout viraux liés à la manipulation du sang et de tout liquide biologique. Ils confondaient par ailleurs les réservoirs animaux des virus et aucun d’eux ne faisait le lien entre les rongeurs et la fièvre Lassa d’une part et entre les chauves-souris et la maladie à virus Ebola d’autre part. Ce constat est d’autant plus inquiétant que Mastomys natalensis est très fréquent au Bénin et surtout au Nord-Bénin où ils envahissent les greniers de conservation traditionnelle des céréales tels le mil et le sorgho. Ces rongeurs et les chauves-souris sont également consommés dans plusieurs localités. En effet, la consommation de Mastomys constitue un facteur de risque de transmission de la fièvre Lassa [13 - 15] alors que la chauve-souris est l’un des principaux réservoirs de la maladie à virus Ebola [16, 17].

Quant aux manifestations cliniques, les trois premiers signes cités étaient : la fièvre, les vomissements et la diarrhée. En réalité ce sont des symptômes qu’ils rencontraient fréquemment chez les enfants de moins de 5 ans. Les moyens de prévention cités par les relais étaient en lien direct avec les voies de transmissions ce qui témoignait d’une certaine logique dans leur réponse. C’était l’éviction des viandes de la brousse, du contact direct avec les malades ou leurs cadavres en cas de décès, et le lavage des mains. Pour ce qui est du comportement des relais dans certaines conditions d’exposition au risque de FHV, seulement 38% disposaient de gants mais ne les utilisaient pas pour examiner les enfants malades. Certains les utilisaient seulement pour faire les TDR mais cela dans une très faible proportion. Aucun d’eux ne disposait des moyens de protection comme : blouse, tablier, solutions hydro-alcooliques, lunettes, masque. Au niveau personnel, 40 % des relais n’observaient pas les mesures de prévention qu’ils connaissaient et sont censés recommander dans leur communauté. Le gap entre la connaissance des moyens préventifs et le comportement réel des individus a été relevé dans certaines études [11, 14]. Devant un cas suspect de FVH, huit relais (13,8 %) l’auraient transporté ou faire transporter sur une moto pour aller au centre de santé contribuant ainsi à la propagation de la maladie. Il est quand même louable de constater que ces relais malgré leur limite en matière de connaissance et de comportement face aux FVH, contribuaient à les prévenir à travers des séances de sensibilisation d’où la nécessité de renforcer leur capacité à travers des formations et des supervisions de proximité.

## Conclusion

Cette étude a permis de constater que les relais communautaires avaient une connaissance globalement acceptable sur la maladie à virus Ebola et la fièvre Lassa. Ils pourraient ainsi contribuer à informer et éduquer leurs communautés sur les moyens préventifs de ces épidémies. Cependant, un gap important existait entre leur connaissance et leurs attitudes préventives. D’où la nécessité de renforcer leurs capacités sur les risques liés aux soins et de leur fournir du matériel adéquat de protection.

### Etat des connaissances actuelle sur le sujet

- Les études évaluant les connaissances, attitudes et pratiques sur la maladie à virus Ebola et la fièvre Lassa s’intéressent souvent aux agents de santé donc à une cible ayant ou supposé avoir des pré-requis en matière de sécurité aux soins et de prévention des ces épidémies graves.

### Contribution de notre étude à la connaissance

- Notre étude à l’avantage de s’intéresser aux relais communautaires faisant fonction d’agents de santé dans leur communauté et qui n’ont pas reçu une formation de base sur la sécurité aux soins ni sur les épidémies;
- Cette étude confirme qu’ils n’ont pas une bonne connaissance du risque lié aux soins médicaux, qu’ils ne disposent pas non plus de matériels de protection adéquats et qu’il est nécessaire de les former et de leur fournir le matériel de protection adéquat;
- Leur formation contribuera aussi à améliorer la qualité des activités de promotion de la santé qu’ils mènent dans leurs milieux respectifs.
